# A Forced Vacation? The Stress of Being Temporarily Laid Off During a Pandemic

**DOI:** 10.1177/07308884221129520

**Published:** 2022-11-30

**Authors:** Scott Schieman, Quan Mai, Philip Badawy, Ryu Won Kang

**Affiliations:** 1 Department of Sociology, University of Toronto, Toronto, Canada; 2 Department of Sociology, Rutgers–The State University of New Jersey, New Brunswick, USA; 3 Department of Sociology, University of Alberta, Edmonton, Canada; 4 University of Toronto, Toronto, Canada

**Keywords:** Job disruption, temporarily laid off, mental health, mastery, COVID-19

## Abstract

A million Canadian workers suddenly became temporarily laid off (TLO) early into the pandemic. How did this affect mental health? Guided by the Stress Process Model (SPM), we would expect that this job disruption should increase psychological distress. However, given the unique context surrounding the early period of the pandemic, we advance the forced vacation hypothesis, which argues that those who became TLO would—at least initially—report lower levels of distress. To address this puzzle, we use a mixed-methods approach combining a national longitudinal survey dataset and in-depth interviews. Our quantitative analyses reveal that individuals who were TLO had lower distress in April 2020 compared with their peers who continued working. Our interviews uncover several potential explanations for these patterns. The findings provide an elaboration to the SPM as the pandemic context altered the meaning of being TLO, making it feel like a “forced vacation”—at least initially.

The initial period of the COVID-19 pandemic—April and May of 2020—triggered job disruption. The economic shocks led to roughly a million Canadians being temporarily laid off (TLO). Given the salience of temporary layoffs in labor market and public discourses, we ask: *How did this pandemic-induced shock affect workers’ mental health?* The Stress Process Model (SPM) offers theoretical insights. Pearlin and colleagues (1981) argued that when a disruptive event occurs, individuals often struggle to re-establish homeostasis (Pearlin and Bierman, 2013; Moen, 2022). A disruptive job event can be classified as a stressor—one that might elevate distress if it “is involuntary, undesired, and unscheduled” (Pearlin et al., 1981:343). Indeed, the U.S. Department of Labor characterizes being laid off as: “one of the most traumatic events you can experience in life” (DOL, 2021). It therefore is reasonable to expect those who were TLO early in the pandemic will report higher distress relative to employees who remained working.

But given the extraordinary context surrounding the pandemic and the temporary nature of the job disruption, we also imagine an alternative view—the *forced vacation hypothesis—*which argues that those who became TLO might report lower distress relative to those who continued working. In his elaboration on the consequences of stress, Wheaton (1999) emphasized the importance of *context* in shaping stress exposure and altering their meaning and threat level. We elaborate on why the context of the pandemic raises potential qualifiers to being TLO which might have caused individuals to appraise this experience as not merely a stressor, but also a “vacation”—at least initially. Using data from the *Canadian Quality of Work and Economic Life Study* (C-QWELS), we did not find evidence to support SPM predictions. In April 2020, we found that workers who were TLO reported less distress than those who remained working. By May 2020, that distress gap vanished. Being TLO was not associated with higher distress in April and May—a pattern that contradicts the SPM.

Why did workers who were TLO not report more distress than their peers who continued working? To reconcile this puzzle, we propose an approach that uses quantitative and qualitative evidence to articulate a *forced vacation* thesis. This perspective argues that the pandemic represents a rare context in which the SPM's predictions might not manifest as expected. Specifically, the experience of being TLO—at least early in the pandemic—unfolded in conditions that potentially buffered against adverse mental health outcomes. First, many were afforded a *transient* pause from work and exempted from work-related stressors. Second, the normativity of being TLO allowed workers to appraise job disruption as part of a societal-level phenomenon affecting the entire market, rather than due to personal qualities or characteristics. Third, financial strain associated with being TLO was alleviated by several factors: increased personal savings and reduced spending, government support, and partial employer-provided pay and benefits. Taken together, these dynamics suggest that many workers experienced their temporary employment interruption as a mandatory and unforeseen “vacation”—a temporary break that would shield them from work-related stressors that those who continued to work endured and provide opportunities to recharge.

Our study makes several contributions. The fact that job disruption is not related to a widened distress gap in the early months of the pandemic is inconsistent with the SPM. In this paper, we document this puzzle and offer ideas to solve it. With a mixed-methods approach, we use data from a unique national survey that contains a pre-pandemic baseline with follow-ups in the early months of the pandemic and then integrate data from in-depth interviews. While our quantitative analyses provide evidence about the initially negative and then null association between being TLO and distress (relative to those who remained working), our qualitative analyses provide narratives about potential reasons underlying such patterns. Our study also adds to a growing literature on nonstandard employment and well-being during the pandemic.

## Background

### Sudden Shock and the Dramatic Rise in Temporary Lay Offs

In February 2020, 19.2 million Canadians had a job or business. During the previous twelve months, the unemployment rate held steady between 5.4 to 5.9 percent. Then, in March of 2020, federal and provincial governments declared states of emergency and implemented measures in response to the pandemic that included the closure of non-essential businesses, restricted travel, and social distancing (Statistics Canada, 2020a). Reductions in economic activity sent jolts throughout the labor market. From February to March 2020, the unemployment rate increased to 7.8 percent—the largest one-month increase since data collection began in 1976. Temporary layoffs/furloughs represented the largest upsurge in unemployment—increasing by 36.4 percent from February. The employment rate was 58.5 percent—the lowest employment rate since April 1997. Roughly 1.3 million Canadians were away from work for the week of March 15 to 21, most likely because of COVID-19 (Statistics Canada, 2020a).

The initial effect only partially captures the blow. If March 2020 was a strong tropical storm, April 2020 was a Category 5 hurricane. By then, a full shutdown was in effect across all provinces and territories (Statistics Canada, 2020b). Employment fell by nearly two million in April—comprised of 1,472,000 full-time workers and 522,000 part-time workers. The unemployment rate increased to 13.0 percent—an increase of 5.2 points in a one-month period and the second highest on record. The depth of the downturn was staggering—with employment dropping 15.7 percent in one month, far surpassing previous downturns (Lemieux et al., 2020).

According to the April 2020 Labour Force Survey, 97 percent of newly unemployed Canadians were classified as “temporary layoffs,” implying that these workers expected to return to their former employer—presumably as the restrictions and lockdown measures were lifted (Statistics Canada, 2020b). The total number classified as temporary layoffs reached an all-time high of 1.2 million Canadians. Traditionally, the proportion of the unemployed population represented by TLO workers has been trivial. The pandemic reversed this trend and established the TLO as a substantial constituent of the unemployed (Statistics Canada, 2020c).

### The Stressfulness of Being Temporarily Laid Off in Context 

The SPM has been a prominent theoretical guide for researchers analyzing the association between job disruption and mental health (e.g., Burgard, Brand, & House, 2007). According to the SPM, a sudden change in employment represents a potential stressor. One way that job disruption may harm mental health is by eroding psychosocial resources, like the sense of mastery (Pearlin et al., 1981). Involuntary job disruption—particularly when prolonged—challenges mastery because it suggests that efforts to avoid or resolve this problem are inadequate. Therefore, we would expect that being TLO should threaten mental health at least partly because of an erosion of mastery. The literature on the scarring effect of unemployment provides ample evidence on the mental health consequences of involuntary employment termination (Burgard, Brand and House, 2007; Ezzy, 1993; Farré, Fasani and Mueller, 2018; Mossakowski, 2009; Murphy and Athanasou, 1999; Olesen et al., 2013; Viinamäki, Koskela and Niskanen, 1996; Vinokur et al., 2000).

But that theoretical and empirical background is juxtaposed against a unique social and economic context: a global pandemic. Here, we apply Wheaton's (1999) ideas about the “context hypothesis” to articulate how the conditions surrounding the pandemic may have changed the meaning of being TLO and the ways that individuals appraise this status—reducing its potency as a stressor. In this alternative view, we advance the *forced vacation* hypothesis: the temporary pause of the work role might have felt—at least initially—like a brief hiatus and therefore contributed to lower distress relative to those who remained engaged in the work role.

Several processes might contribute to why those who were TLO might have initially had less distress. First, akin to a vacation, the TLO classification itself suggests a *temporary* pause in the work role. This resonates with our data. In April 2020, 61 percent of TLO participants said it was “very likely” they would return to their job, 19 percent said “somewhat likely,” 9 percent said “not too/not at all likely,” and 11 percent were unsure. The optimism started to soften in just one month. By May 2020, the percentage who perceived it was “very likely” they would return to their job dropped to 53 percent, 23 percent said “somewhat likely,” 11 percent said “not too/not at all likely,” and 13 percent were unsure. Any initial distress gap might have evaporated once the layoff started to feel less temporary. Another thread of the “vacation” theme is that the TLO were no longer exposed to work role demands—demands that their working peers had to endure during a pandemic. As a parallel, during an actual vacation, one might enjoy a two-week “breather”—relief from stress and time to rejuvenate. In the absence of work, laid-off individuals might have dedicated newfound time to self-care or time with loved ones; during lockdowns, there was little else to do outside of the home.

Second, TLO was a normative status—with approximately 1.2 million Canadians being TLO by April 2020 (Statistics Canada, 2020b). According to social comparison theory (Festinger, 1954), comparisons to similar others might have normalized the experience. The TLO might not have internalized the disruption as a personal deficiency because they could contextualize it as a common phenomenon throughout the labor market. Moreover, the pandemic potentially facilitated downward social comparisons with others who were perceived to not be faring as well—especially with the media deluge about the devastating toll of COVID-19.

Third, job disruption typically reduces income and increases financial strain. But several countervailing factors might have mitigated that fallout: (1) the federal government provided the Canada Emergency Response Benefit (CERB) to workers who were unable to continue working due to COVID-19; (2) some employers kept workers on the payroll and maintained benefits; (3) some were able to tap personal savings; and (4) lockdowns and social restrictions meant fewer opportunities to spend money. Collectively, these countervailing forces might have reduced financial strain that, in turn, should have—according to the SPM—contributed to more distress.

Employees who continued to occupy the work role during the tumultuous months of April and May 2020 remained exposed to usual job demands—but also likely encountered new ones, including the possibility of infection for employees in public-facing jobs or the abrupt adjustments for employees required to work from home. In his articulation of the SPM, Pearlin (1983) identified role restructuring as one of the most problematic role strains that workers encounter—and the ambiguity that accompanies it. During the early months of the pandemic, restructuring and ambiguity were undoubtedly pervasive as organizations navigated new realities caused by the pandemic and government mandates. These ideas suggest that part of the distress gap might be due to the job stress among individuals who continued to occupy the work role.

**Figure 1.**
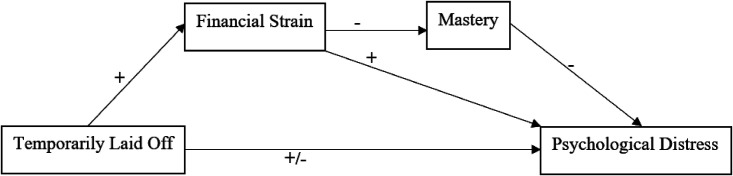
Conceptual framework of the hypothesized associations. *Note*: The “ + /-” sign indicates competing hypotheses.

Collectively, the context of the pandemic provides a unique opportunity to re-evaluate predictions of the SPM. As an elaboration to that model, our *forced vacation* hypothesis might help explain the paradoxical pattern whereby the TLO did not exhibit higher distress. Figure 1 provides a framework to guide our evaluation of the interrelationships.

## Study 1: The Quantitative Portrait

### Sample

To test our hypotheses, we pooled data from two surveys that are part of the *Canadian Quality of Work and Economic Life Study* (C-QWELS). These surveys were intended to examine the quality of work life and well-being among Canadians who were currently employed. Data were gathered in cooperation with Angus Reid Global, a survey research firm that maintains an ongoing national panel of Canadians. All participants are members of the Angus Reid Forum (ARF). The ARF is Canada's most recognized online community of adults managed by an industry-leading team of market research and panel experts.

Sample selection started with creating a balanced sample matrix of the Canadian population. Randomized samples of ARF members were then selected to match this matrix to ensure a representative sample of working adults. The first C-QWELS (I) was conducted from September 19-23, 2019 (N = 2,524). The second C-QWELS (II) was conducted from March 17-23, 2020 (N = 2,528). The first follow-up of all participants occurred from April 17–23, 2020. From C-QWELS I, 1,869 (74%) were successfully recontacted; from C-QWELS II, 2,046 (81%) were successfully recontacted. Then, from May 17-24 2020, we launched the second follow-up with all participants. From C-QWELS I, 1,807 (72%) were successfully recontacted. From C-QWELS II, 1,886 (75%) were successfully recontacted. We apply weights based on census data for the working population according to gender, age, and region.

We took several steps to create the analytical sample. From the pooled baseline sample of 5,052, we removed the self-employed (n = 541). For analyses of the April 2020 sample, we retained those who continued to work or became TLO; we removed 1,232 individuals who were no longer working for some other reason or if they transitioned to self-employment. We removed 94 cases with missing values on study variables. This provided an analytical sample of 3,169 cases for April 2020—with 309 TLOs. Then, for analyses of May 2020, we further retained only those who continued to work or were TLO; this removed 632 cases yielding an analytical sample of 2,537 cases for May 2020—with 355 TLOs.

### Focal Measures

*Psychological distress.* We use five items to measure *psychological distress* (Badawy & Schieman, 2020). The items ask, “In the past month, how often did you feel…”: “anxious or tense,” “nervous,” “restless or fidgety,” “sad or depressed,” and “hopeless.” We coded response choices: (1) none of the time, (2) a little of the time, (3) some of the time, (4) most of the time, and (5) all of the time. We averaged responses to create the index (SEP_α_ = .89; MAR_α_ = .86; APR_α_ = .88; MAY_α_ = .89).

*Temporarily laid off*. In April and May 2020 surveys, we asked participants if they were currently not working because they were TLO or furloughed.

*Financial strain*. We measured financial strain with three items (Bierman, Upenieks, Glavin, & Schieman, 2021). Respondents were asked how often they experienced “trouble paying the bills” and “not have enough money to buy food, clothes or other things your household needed.” We coded response choices: (1) never, (2) rarely, (3) sometimes, (4) often, and (5) very often. A third item asked: “How do your finances usually work out by the end of the month?” We coded response choices: (1) a lot of money left over, (2) a little money left over, (3) just enough to make ends meet, (4) barely enough to get by, and (5) not enough to make ends meet. We standardized the items (because of different response choices) and averaged them to create the index (SEP_α_ = .85; MAR_α_ = .84; APR_α_ = .80; MAY_α_ = .83).

*The sense of mastery*. We measured mastery with four items adopted from the Pearlin and Schooler (1978) scale. Participants were asked the extent they agree or disagree with the following statements: “You have little control over the things that happen to you,” “There is really no way you can solve some of the problems you have,” “You often feel helpless in dealing with the problems of life,” and “Sometimes you feel you are being pushed around in life.” We coded response choices: (1) strongly agree, (2) agree, (3) disagree, and (4) strongly disagree, and averaged the responses to create index (SEP_α_ = .83; MAR_α_ = .81; APR_α_ = .83; MAY_α_ = .83).

*Sociodemographic and work characteristics.* We used dummy-codes to measure *gender* (men = 0, women = 1, non-binary = 2), *visible minority* (non-minority = 0, minority = 1), *marital status* (married = 1, not married = 0), and *presence of children younger than 18 at home* (0 = no, 1 = yes). *Age* was measured in years. For *education*, we compared those with an undergraduate degree or higher to those with less than an undergraduate degree. For *household income*, we compared under $50,000 to $50,000 - $99,999, $100,000 - $149,999, and $150,000 or more. For *occupation*, we compared professionals to clerical, service/sales, and labour/production. We controlled for whether respondents worked in the *private/for-profit* sector, whether they were *salaried*, whether they belonged to a *union*, whether they experienced *job insecurity*, and *job tenure*. For *work hours,* we compared those working 40–49 h to those working fewer than 30 h, 30–39 h, or 50-plus. For *gender composition*, we compared those in workplaces with 51–75% women to the following: 0–25% women, 26–50% women, and 76–100% women. Finally, we adjusted for *region* by contrasting participants in Ontario to Atlantic, Quebec, Manitoba, Saskatchewan, Alberta, and British Columbia. Online Appendix Table A provides odds ratios to display how baseline variables are associated with the probability of being TLO.

## Results

### Analyses of the April 2020 Survey

*Financial strain.* In model 1 of Table 1, financial strain is the dependent variable in analyses of the April 2020 sample. Being TLO is not associated with increased levels of financial strain, net of baseline financial strain and control variables. This finding does not align with a core prediction of the SPM—that job disruption should increase financial strain. At least initially, those who were TLO did not experience elevated financial strain.

**Table 1.** Temporarily Laid Off and its Impact on Financial Strain, Mastery, and Psychological Distress (April 2020)

	Financial Strain	Mastery		Distress		
	Model 1	Model 2	Model 3	Model 4	Model 5	Model 6
Temporarily Laid Off	.082	−.008	.001	−.082*	−.090*	−.081*
Financial Strain			−.096***		.108***	.050**
Mastery						−.401***
Women	.044	−.001	.008	.112***	.102***	.106***
Visible Minority	.136***	−.052†	−.039	−.016	−.034	−.062†
Age	.000	.003**	.003**	−.005***	−.006***	−.006***
Married	−.013	.016	.012	.028	.031	.028
Have Children at Home	.048†	.005	.023	.035	.013	.014
BA Degree or More	−.008	.008	.004	.009	.014	.017*
Household Income (REF = < $50,000)						
$50,000−$99,999	−.050	.016	.000	−.012	.008	.026
$100,000−$149,999	−.066	.048	.020	−.112**	−.078†	−.046
$150,000 or More	−.087	.094*	.055	−.048	.000	.059
Occupation (REF = Professionals)						
Clerical	−.009	−.015	−.016	.025	.025	.016
Service and Sales	.057	−.001	.012	.046	.032	.044
Labour and Production	.076†	.037	.042	−.027	−.035	−.033
Private for−profit	−.021	−.032	−.034	.072*	.073*	.045†
Salaried	−.057†	.005	−.004	.027	.038	.048†
Unionized	.016	−.047†	−.040†	.116***	.108***	.085**
Work Hours (REF = 40–49 h)						
Fewer than 30 Hours	.072	−.046	−.043	.063	.059	.044
30–39 Hours	−.014	.002	−.001	−.006	−.004	−.010
50 or More Hours	.066	.033	.044	.074†	.060	.071†
High Job Insecurity	.150***	−.064*	−.044†	.033	.009	−.032
Gender Composition (REF = 51–75%)						
0–25%	−.010	−.016	−.015	−.008	−.009	−.015
26–50%	.065*	−.026	−.020	.041	.034	.031
76–100%	−.065†	−.022	−.026	−.059†	−.052†	−.046
Job Tenure	.019*	−.007	−.007	−.001	−.002	−.010
Region (REF = Ontario)						
Atlantic	−.018	.019	.020	−.005	−.008	−.008
Quebec	−.113**	−.005	−.021	−.026	−.011	−.034
Manitoba	.079	−.071	−.058	.076	.062	.041
Saskatchewan	.092	−.024	−.008	.001	−.019	−.027
Alberta	.017	−.074*	−.074*	.009	.009	−.031
British Columbia	.028	−.004	−.008	.011	.015	.012
Baseline Financial Strain	.541***					
Baseline Mastery		.659***	.628***			
Baseline Distress				.665***	.638***	.483***

*Note*: This table reports unstandardized regression coefficients derived from OLS models. †p < .10. *p < .05. **p < .01. ***p < .001.

*Mastery.* Model 2 of Table 1 shows that being TLO is not significantly associated with mastery in April 2020. Those who were TLO did not experience lower mastery compared to individuals who remained working. In model 3, we include financial strain and show it is linked to lower mastery (b = -.096, p < .001). Moreover, the TLO coefficient remains statistically non-significant. Taken together, except for the association between financial strain and lower mastery, these initial patterns do not support SPM predictions. At least in April, individuals who were TLO did not experience lower mastery relative to their peers who remained working.

*Distress.* Model 4 of Table 1 shows that individuals who were TLO in April 2020 had *lower* distress relative to those who continued working (b = -.082, p < .05)—and this manifests net of baseline distress and controls. In models 5 and 6, respectively, financial strain is associated with higher distress (b = .108, p < .001) and mastery is associated with lower distress (b = -.401, p < .001). However, adjusting for financial strain and mastery has little influence on the TLO coefficient (b = -.081, p < .05). As a sidenote, the coefficient for financial strain decreases by 53.7 percent across models 5 and 6 (b = .050, p < .01). One way to interpret this is that part of financial strain's positive association with distress operates indirectly through its negative association with mastery.

Taken together, our findings do not support the SPM's proposition that job disruption increases distress. Instead, we find the opposite pattern: those who were TLO in April 2020 reported lower distress relative to their peers who continued working. In the SPM, Pearlin and colleagues (1981) observed that elevated financial strain and reduced mastery contributed to elevated distress among those experiencing job disruption—and that helped to explain why those individuals experienced more distress. But we found no evidence of increased financial strain or lower mastery among those who were TLO in April. These patterns imply that conventional takes on the explanatory role of financial strain and mastery are not applicable in this scenario.

Is it possible that our patterns reflect a dynamic in April 2020 that is more akin to a *forced vacation*? Wheaton's (1999) context hypothesis directs us to consider the early months of the pandemic as a force that might have altered the meaning of being TLO—in ways that made it feel less like a conventional job disruption stressor and perhaps more like a short-term breather. But that seemed to change by May of 2020.

### Analyses of the May 2020 Survey

*Financial stress.* Turning to May 2020, model 1 of Table 2 shows that being TLO is now significantly associated with elevated financial strain (b = .105, p < .05). This is an important difference from the non-significant association observed in the April analyses. The elevated financial strain among the TLO is consistent with the SPM's predictions—and is suggestive that perhaps the initial sense of being on a “vacation” of sorts started to wear off by May.

**Table 2.** Temporarily Laid Off and its Impact on Financial Strain, Mastery, and Psychological Distress (May 2020)

	Financial Strain	Mastery		Distress		
	Model 1	Model 2	Model 3	Model 4	Model 5	Model 6
Temporarily Laid Off	.105*	−.068*	−.049	−.008	−.028	−.038
Financial Strain			−.128***		.151***	.075***
Mastery						−.463***
Women	.029	−.003	.008	.083**	.071*	.078**
Visible Minority	.143**	−.068*	−.048	−.052	−.085*	−.121**
Age	−.001	.004**	.004***	−.005***	−.006***	−.006***
Married	.005	.025	.025	−.002	−.004	.000
Have Children at Home	.025	−.018	.003	−.007	−.036	−.053*
Bachelor's Degree or More	−.010	.017*	.011	−.006	.002	.009
Household Income (REF = < $50,000)						
$50,000 to $99,999	.029	.032	.023	.002	.016	.046
$100,000 to $149,999	−.030	.010	−.021	−.136**	−.094†	−.071†
$150,000 or More	−.083	.087†	.036	−.057	.010	.075
Occupation (REF = Professionals)						
Clerical	.016	.003	.006	−.015	−.018	−.011
Service and Sales	−.014	.004	.015	−.031	−.043	−.026
Labour and Production	.044	.042	.046	−.103*	−.110*	−.100*
Private for−profit	−.007	.005	.002	.049	.049	.036
Salaried	−.058†	.011	−.000	.019	.035	.051†
Unionized	−.030	−.026	−.024	.118**	.115**	.096**
Work Hours (REF = 40–49 h)						
Fewer than 30 Hours	−.001	−.078†	−.085*	.061	.068	.023
30–39 Hours	−.031	−.011	−.017	.017	.022	.005
50 or More Hours	.050	−.053	−.045	.057	.046	.015
High Job Insecurity	.218***	−.095**	−.058†	.110**	.063†	.016
Gender Composition (REF = 51–75%)						
0–25%	−.001	.016	.020	−.040	−.045	−.040
26–50%	−.018	−.016	−.020	−.002	.003	−.001
76–100%	−.104*	.043	.030	−.066	−.048	−.019
Job Tenure	.032**	−.010	−.008	−.000	−.005	−.014
Region (REF = Ontario)						
Atlantic	−.003	.035	.038	−.079†	−.084†	−.068
Quebec	−.069†	−.029	−.042	−.039	−.025	−.050
Manitoba	.040	.014	.025	−.037	−.047	−.015
Saskatchewan	.085	−.027	.000	−.020	−.053	−.052
Alberta	−.025	−.068*	−.076*	−.048	−.040	−.079*
British Columbia	.055	−.052†	−.051†	.041	.040	.020
Baseline Financial Strain	.545***				
Baseline Mastery		.623***	.585***			
Baseline Distress				.648***	.611***	.433***

*Note*: This table reports unstandardized regression coefficients derived from OLS models. †p < .10. *p < .05. **p < .01. ***p < .001.

*Mastery.* Model 2 of Table 2 shows that being TLO is significantly associated with lower mastery in May 2020 (b = -.068, p < .05)—another divergence from what we observed one month prior. This pattern aligns with the SPM's predictions about the psychosocial consequences of job disruption. Moreover, it supports the idea that as the experience of being TLO pressed on, this might have started to erode individuals’ sense of mastery. Adding financial strain in model 3, which is associated with reduced mastery (b = -.128, p < .001), helps explain why the TLO report lower mastery relative to their peers who continued working. Across models 2 and 3, the absolute size of the TLO coefficient decreases by 27.9 percent and is no longer statistically significant. Taken together, these interrelationships align with core predictions of the SPM.

*Distress.* Model 4 of Table 2 shows that being TLO is not significantly associated with distress in May 2020; there is no longer any difference in distress between those who were TLO and their peers who continued working. While financial strain (b = .151, p < .001) and mastery (b = -.463, p < .001) both predict distress, the lack of a distress gap holds. Moreover, as we noted above, about 50.3 percent of the association between financial strain and distress observed in model 5 is explained by the inclusion of mastery in model 6. These interrelationships are consistent with the SPM.

Taken together, what do these findings suggest about the veracity of the SPM? The portrait is mixed. On one hand, the fact that we do not find elevated distress among the TLO contradicts the SPM. However, there is also evidence that any “honeymoon phase” of a forced vacation observed in April 2020 started wearing off by May—especially in terms of elevated financial strain and reduced mastery. Next, we turn to qualitative analyses to flesh out that story.

## Study 2: The Qualitative Portrait

### Sample and Data Analysis

The qualitative part of our paper asks: What processes contribute to the lack of elevated distress among individuals who are TLO? To gain insights on the *forced vacation* thesis, we conducted in-depth interviews with workers who were part of the TLO subsample in the C-QWELS. When the pandemic hit, many reported that they were TLO in our surveys conducted in April and May 2020, so we interviewed 47 of these respondents from January to June 2021. We developed an interview guide that probed a range of experiences associated with being TLO. In-depth interviews are ideal to probe how individuals interpret events in social life and unpack complex processes that underlie the nuances of experiences and perceptions (Orbuch, 1997). Using NVivo, the authors separately analyzed interview transcripts, developed nodes to code themes, met frequently to discuss themes, and continued to review transcripts and refine themes.

All interviewees were employed at baseline and became TLO in April or May 2020. Interviews were conducted either by Zoom or phone and ranged from 35 to 75 min. Online Appendix Table B lists the gender, age, industry, and province of participants: 48.9 percent are women; 25.5 percent are visible minorities; and age ranged from 25 to 62. The sample is geographically diverse: Ontario (40.4%), British Columbia (34.0%), Alberta (12.8%), Nova Scotia (4.3%), Saskatchewan (4.3%), New Brunswick (2.1%), and Manitoba (2.1%). Participants held jobs in a broad and diverse range of occupations and industries. To ensure anonymity, we used pseudonyms and, in some cases, described job titles or industries in more general terms.

### Findings

#### A Forced Vacation

Many employees evoked the language of “taking a break,” “relaxing,” and “vacation” while discussing the job disruption. After receiving news of the temporary layoff, Paula recalled feeling relieved: “There was honestly a certain level of relief that I got a break, because I have been working steady without a vacation for a while.” Vanessa, an educator from Ontario, mentioned that when she found out about being laid off, she decided to “pack up and go to the cottage, and I stayed there from March to the beginning of June. So, I took a mini vacation.” Likewise, Adam described his experience as follows:

“It was just like a *break* […] like a *temporary break* […] We were pretty well going six days a week so didn't really mind the […] like *extra vacation*. You get used to doing the six days a week because human nature wants that extra money, right? [Laughs]. And then it just kind of stops and you take a deep breath and say, ‘Well, just sometimes the money is not worth it.’”

Rose, a warehouse clerk from British Columbia, explained that after getting laid off, she just “stayed home,” “did not have much worry,” and was able to “treat it as a short vacation.” Echoing the point, Calvin explained:

“Even if I’d wanted to, there was nothing I could have done at that point. But at the same time, taking away that option was nice, I kind of got to kick my feet up and enjoy doing some other stuff. […] Sometimes not having the option is also kind of nice because it *forced my hand* and *I didn’t have a choice*, so I just got to, *forced me to relax a little bit*, just try to enjoy it I guess as best I could.”

Earl explained that after he was notified about the layoff, he found himself in a “strange position” where his work “just stopped.” He pondered: “Now what am I going to do? It's like being on a *forced vacation*” [emphasis ours]. Phrases like “forced my hand” and “didn’t have a choice” illustrate workers’ perception that the pandemic represented an event that was beyond individual-level control.

Given the common accounts of a “forced vacation,” we wondered: How, if at all, did that perspective have a protective effect—at least in the short-term—on mental health? The qualitative data revealed three possible reasons why the “forced vacation” perspective might have contributed to lower distress levels among the TLO. First, workers expected their TLO status to be just that: *temporary*. During the pause, unlike employees who continued working, those who were TLO were no longer exposed to work-related stressors. Instead, many said they could dedicate newfound free time to regenerative activities like self-care or connecting with family. Second, the normativity associated with being laid off during the pandemic contributed to workers’ tendency to attribute job disruption to structural rather than personal factors. Third, the TLO were able to mitigate or avoid financial strain that is typically associated with job disruption. The following sections provide evidence for these themes and their protective effects.

[1] A Temporary State: expectations to return, exempted from work stress, and time for self-care

*Expectations about returning to work.* Most participants expected their TLO status to be temporary. Akin to an extended vacation, workers did not expect the pause to be permanent. Jennifer observed:

“It really felt… *temporary*, *it felt like this was just a delay* [emphasis ours]. Once everything gets settled, and sorted out, this isn’t going to last long. We’ll all be back to work soon, and it really felt like that […] then as time progressed and numbers rose, it really felt like it might not happen. But I never felt like they weren’t going to bring us back.”

Sharing Jennifer's sentiment, Ashley asserted: “I think everybody expected it to not last long. I don’t think anybody thought it would have lasted as long as it had.” Other participants stated that despite not having knowledge about how severe the pandemic would become, they expected to return to work after a break. Charlie said: “I had a feeling it was temporary. We really didn’t know how long this pandemic was going to go […] I just figured it was temporary.” Shawn expected the disruption to be limited because of his seniority: “So immediately after the layoff I was like, ‘Okay, I'm totally confident I'm going to get my job back within 3 months.’ I'm quite high in seniority of this department. So, I wasn’t terribly worried.”

At this juncture, it is worth underlining a tenet of the SPM. According to Pearlin et al. (1981), a disruptive event creates a disequilibrium and imposes a period of adjustment during which the system attempts to re-establish the default state. Stress arises during this attempt at readjustment. It is reasonable to assume that when participants perceived a relatively short period of adjustment, and homeostasis could be predictably regained, they would be less vulnerable to stress. A temporary disruption would not have the same jolt as an enduring or chronic stressor. Shawn's assertion of confidence that he would return to his job within three months—and its link to not being “terribly worried”—reflects this dynamic. Expectations about returning to work, therefore, seemed to provide some protection against more injurious appraisals of the disruption.

*Exemptions from work-related stress.* Being TLO gave workers a break from exposure to work-related stressors, and this represents another potential protective factor during the emerging months of COVID-19. Many participants described their work conditions just prior to the pandemic as demanding and stressful. Within that context, it is reasonable to interpret a pandemic-induced break as a welcomed “breather”—like a vacation. Peter observed:

“My work was quite stressful. […] It was always high pressure, like you’re always getting squeezed to do too much. […] The stress from work was weighing on me pretty heavy. And [after getting laid off], *I don’t have that anymore, which has been absolutely awesome*. […] *It was less stress.”*

Peter linked the relief from work stress within the TLO status as reducing distress. A common theme from the interviews centered on this sense of relief from heightened job stress prior to the layoff. Danielle, an airline sales representative, explained:

“Honestly, I was really happy to be laid off because the whole month of March, everything we dealt with was escalations, people freaking out, trips being cancelled, nobody getting refunds. I'm telling you the day I got my layoff I was kind of *happy to not have to show up to work anymore* [emphasis ours]. […] I was stuck at home, whatever, it's fine. I'm not catching the coronavirus. Some people had to go to work and risk their lives. I was lucky enough to get the chance to not have to do that.”

As these quotes suggest, March of 2020 was stressful as societal and economic changes surfaced. Workers were overwhelmed by demands and received little support from short-staffed teams. Danielle mentioned that she did not have any supervisors, so she felt relieved when she was laid off. Her experience exemplifies how a variety of factors potentially protected workers from distress: the forced vacation, the break from work, the avoidance of work-related stress, and the safety of not being exposed to the virus. Echoing Danielle's account, Janet explained:

“When the wage subsidy program got hashed out by the government and my company ended up qualifying for it, I then started getting the 75% of my pay, but I didn’t have to go back to work. […] The rest of my co-workers that were working […] were like, “We’re only making 5% more than you, and we’re still working our asses off!” At that point, I was glad I was in the furloughed group because I don’t know if I would’ve wanted to deal with *the stress of trying to keep the lights on*” [emphasis ours].

Danielle and Janet articulated how demanding those early weeks of the pandemic were—and a sense of relief being spared. We see these narratives as potentially contributing to why the TLO did not report higher distress than those who remained engaged in the work role. Emerging scholarship on work during the pandemic indicates that many experienced an increased workload, health and safety concerns, and scheduling adjustments (Said and El-Shafei, 2021). For the TLO group, not being exposed to these novel stressors may have had a protective effect.

*Self-care and family time.* While job disruption is typically a distressing experience, our participants reported that during the pandemic-induced layoff, they had time to connect with family and engage in self-care. Akin to a vacation, a short break from work allowed participants to reconnect and recharge. When the drilling rig where he worked was shut down, Ben was TLO from his position as a loader operator. When we asked how stressful the experience was, he replied: “I would have said ‘not at all.’ I get along good with my girlfriend, I actually love being home with her. It's nice. A month off is no big deal, right?” Similarly, after she was laid off from her job in the food service industry, Corinne found time to fulfill her new year's resolution: To spend more time with family. Being laid off afforded Corinne the opportunity to go to picnics with her cousins’ spouses and children:

“I did get to spend more time with family, [and it was] wonderful. And I learned about them, and they learned about me, and it was, rather than just the aunt that comes for a day or two every once in a while.”

Peter also noted the newfound time for family, something his busy work schedule did not allow:

“I get to spend more time with the kids which I have been enjoying. It's more fun to hang out with them here and there, just take an afternoon to take them sledding. You know, I had some weeks [when I worked] 60 or 70 h. I wake up, eat breakfast, go to work, come back, see the kids, pat them on the head, they go to bed, you go to bed shortly thereafter. So, it's been nice to be around them.”

Similarly, Rose's relationship with her family improved while she was laid off. She received more frequent communication from her daughters, who lived far away: “Although we cannot see each other, we talk much more than before. They call three or four times a week. Then before, they only called us once a week. They care about us, and our relationship got closer than before.”

Katrina characterized being laid off as an “opportunity to step back and take care of yourself, just to focus on you and your body, your health, your everything.” She said it was a “blessing” because she “learned so much not just about [herself], but also about her husband and being together for three months constantly.” She described how their relationship improved:

“We never spent as much time together off work, so it was a little bit weird at the beginning constantly being home and seeing each other […]. I just feel like *this relationship got even better*! The communication's amazing, the way how we are able to discuss things and the trust […] it's amazing” [emphasis ours].

The boost that some participants received came from beyond their immediate family. They also reported that being away from work allowed them to have better relationships with friends and neighbors. Katrina mentioned that since she immigrated to Canada, she has been constantly busy with the ongoing cycle of work. The break from work allowed her to rekindle connections:

“Now, during the pandemic, you had the time. You were able to actually create more bonds and able to deal with them on a daily basis, to call them, video call, this and that. Same thing with friends. There were people who I haven’t been in touch with for years and months, and now suddenly you get that opportunity to be able to talk to them, so that was a big plus. I really enjoyed that I was able to stay in touch and make those friendships and family relationships even stronger.”

Connecting with family and friends was easier when participants were not engaged with work. Being laid off afforded time to access and build supportive bonds. These processes might have been more challenging for workers who remained in full-time jobs during the early months of the pandemic, as work demands restrict time and attention required for self-care and family time.

[2] Reduced Internal Attribution

In April 2020, roughly 97 percent of the newly unemployed were classified as TLO (Statistics Canada, 2020b). Being TLO was therefore a widespread structural phenomenon, one that affected a substantial swath of the labor market. It is reasonable then to suspect that job disruption was more broadly perceived as attributable to structural circumstances and not personal attributes or actions. When we asked Darlene if being laid off affected her sense of self, she responded:

“Not really, no. I’ve seen so many people get laid off, it wasn’t like they [the employer] picked on […] a certain age range to let go. […] I know they weren’t being biased. *They were doing what they had to do to keep their heads above water*” [emphasis ours].

Rebecca reiterated Darlene's sentiment: “it was through no fault of your own that you’ve lost this job.” Others echoed on family members’ attributions. As Frank put it: “My wife says it's not my fault […] I know it's not my fault.” Similarly, some saw job disruption as a common phenomenon affecting workers across broad swaths of their particular industries. Calvin explained that the pandemic “put a wrench on our entire industry,” and there was nothing that he could have done to prevent the situation, attributing being TLO to actions of powerful others—he felt the government lockdowns and restrictions were implemented in ways that undercut his industry. Similarly, Mitchell, a geological technician, explained:

“It did feel like a break. Like I was back in […] high school and this is my summer off school and […] we didn’t look at our day-to-day life the same we did as when I was working. It was more like […] not worrying about what's going to happen because you know, *I was going through the same things a lot of my friends and peer groups were going through and I could see some of them were struggling even worse than I was*. So, we felt kind of blessed that we were able to not change our lifestyle drastically” [emphasis ours].

The fact that so many Canadians were affected—and the circumstances were beyond any individual's control—reduced negative internal attributions. This finding is consistent with theoretical perspectives on how individuals cope with adverse structural forces. Studies on social comparisons maintain that individuals might benefit from collective support by promoting a belief that others undergo comparable experiences—contributing to a generalized sense of a shared common stressor (Clark, 2003; Lin, Ye and Ensel, 1999; Suls and Wheeler, 2013). When the experience is widely shared, workers are less likely to be harmed by the stigma associated with job disruption and less inclined to engage in negative self-appraisals (Glavin and Young, 2017; Young and Wheaton, 2013). Like unemployed workers in areas with high unemployment, those who were TLO were aware that many workers were also enduring similar experiences, so the high rates of temporary layoffs likely normalized it. This awareness might have reduced self-blame and other forms of negative attribution, thus operating as another potential buffer that protected mental health.

[3] Alleviated Financial Strain by governmental support, personal savings, and partial employer-provided pay and benefits

In the SPM, job disruption might generate financial difficulties, which can function as a threatening secondary stressor that leads to mental health problems (Kahn and Pearlin, 2006). However, contrary to that view, some participants reported that they were able to draw on other financial resources—at least in the short-term. In response to the economic damage inflicted by the pandemic, the federal government created several benefit programs and subsidies, such as the Canada Emergency Response Benefit (CERB). In 2020, approximately one third of all workers who had earned at least $5,000 in 2019 received CERB payments (Morissette et al., 2021). Governmental support created a financial buffer for workers. Raymond explained that when he started collecting CERB, he thought: “I got enough coming in to pay the bills; I got enough to keep myself alive and keep my head above water.” The CERB helped alleviate his anxiety: “If you have all these bills to pay and there wasn’t CERB, let's just say it would be totally different. There would be this fear: ‘how am I going to make the mortgage? how am I going to pay for this, for that?’ Charlie reinforced that point: “I wasn’t too worried about it because I knew I could collect CERB. CERB […] helps me covering my debt and buying groceries. It wasn’t a huge stress; it wasn’t a financial stress necessarily.” Others explained that personal savings helped cope with the lack of work-generated income. Vanessa observed: “We had a significant amount of savings which we knew would carry us through for a good portion of time. So, I really took the opportunity to kick back, relax, and isolate.” Some employers also continued to provide partial salaries and benefits to their TLO workers. After being laid off from his architectural firm, Earl relied on a combination of family support, partial salary, and benefits to avoid strain:

“I was getting paid *75% of my salary, I had full benefits* and I was basically paid to do nothing. I’ve ran out of fingers on my hand of how many people I’ve met over the years that that's their ideal job, they would love to be paid to do nothing…which, for the first couple of weeks, it was like vacation and time to adapt. […] We weren’t struggling financially like a lot of other people. And in terms of physical and mental health, there were *no issues*” [emphasis ours].

Eddie, a retail trade salesman, told a similar story:

“At the start of the pandemic, that was almost like the *honeymoon phase*. It was almost somewhat of an exciting time because it was unprecedented, it was just strange. We were *collecting benefits* [emphasis ours], so at that point, like early in the pandemic, *I’d say* ‘*not stressed.*’”

Alongside these supports and savings, many also reported that their spending decreased significantly during the early months of the pandemic. With lockdowns and social distancing, individuals’ discretionary spending decreased. Patricia explained that while she was somewhat anxious about her finances after getting laid off from her job as a flight attendant, she quickly realized: “It wasn’t as negative as I thought it was going to be. I’m actually saving money by not spending as much on dining out and entertainment.” Similarly, Oliver explained: “I am a very social person. I usually throw parties or go to them [but now] I don’t do those things. I traditionally spend most of my disposable income on travel; as you can tell, [now] I don’t do that.” When we asked how the pandemic affected her finances, Paula responded:

“The main reason it wasn’t terrible was that there was absolutely nothing to spend money on. [The balance between] what we were bringing in versus what we were putting out was very manageable. We cancelled one of our cars’ insurance policies and we just didn’t drive it. […] We weren’t going out and seeing friends, doing things, that's a huge chunk of young unmarried people's income. And so, by doing that, it was manageable.”

These quotes demonstrate the countervailing forces that offset the strains from income loss associated with being TLO: personal savings, government support, employers’ partial pay and benefits, and social restriction-induced reduction in discretionary spending.

## Discussion

In light of the dramatic changes to the labor market brought about by the pandemic (see Chavez et al., 2022; Fan and Moen, 2022) and the rising work precarity and its implications for workers’ socioeconomic well-being (Ayala-Hurtado, 2022; Mai, 2021; Gevaert et al., 2021; Mattijsen et al., 2022), it is important to examine how the pandemic-induced job disruption matters for mental health. The SPM provides a framework for examining these dynamics. Following the SPM, we expected that workers who were temporarily laid off would experience more distress relative to their counterparts who continued working. However, the unique context of the pandemic generated countervailing circumstances under which that assumption did not hold. Instead, we discovered a paradoxical finding contrary to SPM's predictions.

With longitudinal data, we demonstrated that those who were TLO in April 2020 had *lower* distress than their counterparts who continued working. While this distress gap disappeared by May 2020, we did not find that the TLO had higher distress—and that pattern runs counter to the SPM. We also found that mastery and financial strain did not operate as mediators or suppressors in those relationships. Alongside the quantitative analyses, we analyzed data from in-depth interviews with participants who were TLO to inductively develop possible explanations for the quantitative patterns. We label our explanation the *forced vacation* hypothesis and identify three themes. First, workers perceived being TLO as a break and expected a short-lived pause rather than a long-term disruption. Many benefitted from that pause by avoiding work-related stressors, finding opportunities to recharge, and giving themselves newfound time for self-care and loved ones. Like a vacation, this pause protected workers from being exposed to the increased stress associated with working through the tumultuous early period of the pandemic. By contrast, those who continued working likely faced role restructuring or increased workloads or other unanticipated pressures related to COVID-19. Second, the TLO attributed their employment interruption to structural factors—not personal ones. Consistent with social comparison theory, the collective experience protected mental health because they were “in the same boat” as others. And this normativity also reduced internal attributions for the job disruption. Third, another protective factor stems from the lack of financial strain—at least in the short-term. Workers reported that they did not experience financial strain because of increased government aid, employer-provided pay and benefits, personal savings, and reduced spending.

There are important qualifiers to these findings and our interpretations. As discussed, many of our respondents equated being laid off to a “forced vacation.” But like a vacation, the protective effects can be short-lived. As a vacation turns into a three- or four-month hiatus, expectations about the temporary nature of the disruption might have waned. Analyses do not include the length of being in the TLO status. But based on the SPM, it is reasonable to assume that those participants who experienced a longer job disruption experienced greater reductions in mastery and more distress. Moreover, some participants noted that some of their peers were recalled before them. That might have further amplified uncertainty in ways that threaten mental health. With respect to financial strain, some offset income loss with personal savings or government support. However, these approaches were considered stopgap measures rather than long-term strategies. Savings eventually run out—as did government support (CERB was suspended after 28 weeks of payments). In sum, while the factors discussed above could have mitigated adverse outcomes in the early phase of being TLO, these factors might have weakened as the pandemic wore on—and as the “temporary” quality shifted to a more chronic state.

## Conclusion

In reflecting on ways our discoveries link to other similar processes in the stress literature, we acknowledge how our “forced vacation” thesis might parallel the literature on “relief events.”^1^ When characterizing Thoits' (1995) findings about this phenomenon, Ungar (2003: 91) observes that a relief event describes the experience when “negative events might actually come with *emotional relief*, such as when a divorce, or death of a loved one who is ill, is *anticipated and welcomed* [emphasis added].” We see at least three ways that “relief events” differ from the “forced vacation” conceptualization. First, anticipation is a key component of a relief event. With the “forced vacation” idea, a worker who is TLO within the context of an unexpected pandemic has a limited ability to anticipate or plan. Second, a relief event typically describes a more permanent experience and offers possible closure. By contrast, the *temporarily* laid off worker—as the classification defines them—expects to eventually return to their original work arrangement. Third, in the relief events literature, there is little uncertainty about the events themselves. Once a loved one has died, there is no possibility that they will come back. After a divorce, family dissociation is not pending. By contrast, as time marches on, the temporarily laid off might be unsure about if or when they will return to their old jobs or, if they do eventually return, how their roles might change. Taken together, these differences—the lack of anticipation, the temporary nature, and the potential for chronic uncertainty—distinguish the “forced vacation” from the conventional “relief event.”

Before concluding, we acknowledge a few study limitations. First, the nature of our sample limits understanding of the most disadvantaged workers in the population—especially those unable to be represented in the ARF panel. Second, Canada has a relatively generous welfare state (in comparison to the United States). Being TLO might be more distressing in a context with fewer governmental supports. Third, our interviews took place after the initial TLO period in 2020; the employment experiences and job qualities in subsequent months might have influenced participants’ recollections. Despite these limitations, our research makes several contributions. We go beyond documenting a phenomenon that is puzzling to the SPM and develop a novel explanation—the *forced vacation* hypothesis—to reconcile it. Our study combines unique quantitative data with rich qualitative data to describe how being TLO shapes well-being. The quantitative component documents the link between being TLO and mental health; the qualitative component details the experiences behind the coefficients, articulating how the *forced vacation* hypothesis might have operated. As we complete this paper in 2022, the pandemic is ongoing—and has changed the ways we think about the role of work. Because of this, sociological knowledge about the pandemic's long-term effects on work and health will remain a ripe area of inquiry for years to come. We hope that our study provides useful conceptual, theoretical, and empirical guideposts for those endeavours.

## Supplemental Material

sj-docx-1-wox-10.1177_07308884221129520 - Supplemental material for A Forced Vacation? The Stress of Being Temporarily Laid Off During a Pandemic Click here for additional data file. Supplemental material, sj-docx-1-wox-10.1177_07308884221129520 for A Forced Vacation? The Stress of Being Temporarily Laid Off During a Pandemic by Scott Schieman, Quan Mai, Philip Badawy and Ryu Won Kang in Work and Occupations

## Notes

1. We thank an anonymous reviewer for bringing up this point.
