# Supplementary material for: A Forced Vacation? The Stress of Being Temporarily Laid Off During a Pandemic
Source: Work Occup. 2022 Nov 30:07308884221129520. doi: 10.1177/07308884221129520 (PMC9742739; doi:10.1177/07308884221129520)
Supplement: sj-docx-1-wox-10.1177_07308884221129520 - Supplemental material for A Forced Vacation? The Stress of Being Temporarily Laid Off During a Pandemic [file sj-docx-1-wox-10.1177_07308884221129520.docx]

| APPENDIX TABLE A. Baseline Measures as Predictors of Being Temporarily Laid Off | | |
| --- | --- | --- |
|  | April 2020 | May 2020 |
| Psychological Distress | 1.241* | 1.227† |
| Financial Strain | .987 | 1.065 |
| Mastery | 1.029 | 1.066 |
| Women | 1.278 | 1.404 |
| Visible Minority | .874 | .893 |
| Age | 1.001 | 1.022*** |
| Married | 1.019 | .782 |
| Have Children at Home | 1.300† | .984 |
| Bachelor’s Degree or More | .976 | .989 |
| Household Income (REF = < $50,000) |  |  |
| $50,000 to $99,999 | .984 | .920 |
| $100,000 to $149,999 | .791 | .851 |
| $150,000 or More | .675 | 1.026 |
| Occupation (REF = Professionals) |  |  |
| Clerical | 1.925** | 1.339 |
| Service and Sales | 2.708*** | 2.559*** |
| Labour and Production | 2.824*** | 2.080** |
| Private for-profit | 2.568*** | 1.609** |
| Salaried | .450*** | .397*** |
| Unionized | .688† | .907 |
| Work Hours (REF = 40-49 hours) |  |  |
| Fewer than 30 Hours | 1.674* | 3.389*** |
| 30-39 Hours | .937 | 1.162 |
| 50 or More Hours | 1.002 | 1.377 |
| High Job Insecurity | 1.208 | 1.751*** |
| Gender Composition (REF = 51-75%) |  |  |
| 0-25% | 1.302 | 1.047 |
| 26-50% | 1.582* | 1.391† |
| 76-100% | 1.576* | 1.271 |
| Job Tenure | .868** | .842*** |
| Region (REF = Ontario) |  |  |
| Atlantic | 1.245 | .769 |
| Quebec | .735 | .419*** |
| Manitoba | 1.241 | .562† |
| Saskatchewan | .851 | .735 |
| Alberta | .943 | .547** |
| British Columbia | 1.104 | .811 |

*Note*: Odds ratios are shown in the table. †p<.10. *p < .05. **p < .01. ***p < .001.

| APPENDIX TABLE B**.** Characteristics of Interview Respondents | | | | |  |
| --- | --- | --- | --- | --- | --- |
| No. | Pseudonym | Gender | Age | Industry | Province |
| 1 | Adam | Man | 61 | Manufacturing | Ontario |
| 2 | Amber | Woman | 29 | Accommodation and food services | Nova Scotia |
| 3 | Ashley | Woman | 43 | Nonprofit | Ontario |
| 4 | Ben | Man | 42 | Natural resources | British Columbia |
| 5 | Calvin | Man | 30 | Accommodation and food services | Ontario |
| 6 | Charlie | Man | 47 | Construction | Ontario |
| 7 | Christine | Woman | 25 | Retail trade | Ontario |
| 8 | Corinne | Woman | 43 | Accommodation and food services | Ontario |
| 9 | Danielle | Woman | 39 | Finance, insurance, and real estate | New Brunswick |
| 10 | Darlene | Woman | 58 | Agriculture | Alberta |
| 11 | Doug | Man | 48 | Manufacturing | Ontario |
| 12 | Earl | Man | 53 | Construction | Alberta |
| 13 | Eddie | Man | 39 | Retail trade | British Columbia |
| 14 | Ellen | Woman | 40 | Natural resources | British Columbia |
| 15 | Erica | Woman | 46 | Health care and social assistance | Saskatchewan |
| 16 | Frank | Man | 54 | Transportation and warehousing | Ontario |
| 17 | Greg | Man | 43 | Educational services | Ontario |
| 18 | Holly | Woman | 40 | Educational services | British Columbia |
| 19 | Irene | Woman | 31 | Public administration | British Columbia |
| 20 | Jake | Man | 28 | Educational services | British Columbia |
| 21 | Janet | Woman | 38 | Educational services | Alberta |
| 22 | Jason | Man | 35 | Accommodation and food services | British Columbia |
| 23 | Jennifer | Woman | 51 | Health care and social assistance | Alberta |
| 24 | Katrina | Woman | 33 | Health care and social assistance | Ontario |
| 25 | Kendra | Woman | 35 | Professional/scientific/technical services | British Columbia |
| 26 | Lawrence | Man | 41 | Information, culture, and recreation | British Columbia |
| 27 | Leonard | Man | 57 | Manufacturing | British Columbia |
| 28 | Linda | Woman | 49 | Finance, insurance, and real estate | Ontario |
| 29 | Marie | Woman | 36 | Transportation and warehousing | Alberta |
| 30 | Meghan | Woman | 58 | Natural resources | Saskatchewan |
| 31 | Mitchell | Man | 32 | Professional/scientific/technical services | British Columbia |
| 32 | Oliver | Man | 36 | Public administration | Alberta |
| 33 | Patricia | Woman | 35 | Transportation and warehousing | British Columbia |
| 34 | Paula | Woman | 29 | Other services | Ontario |
| 35 | Peter | Man | 45 | Agriculture | Nova Scotia |
| 36 | Raymond | Man | 62 | Transportation and warehousing | Manitoba |
| 37 | Rebecca | Woman | 51 | Public administration | British Columbia |
| 38 | Richard | Man | 57 | Transportation and warehousing | Ontario |
| 39 | Robert | Man | 39 | Finance, insurance, and real estate | Ontario |
| 40 | Robin | Woman | 33 | Transportation and warehousing | Ontario |
|  |  |  |  |  | (*Continues*) |
| APPENDIX TABLE B. (*Continued*) | | | | | |
| 41 | Rose | Woman | 58 | Wholesale trade | British Columbia |
| 42 | Ryan | Man | 30 | Health care and social assistance | Ontario |
| 43 | Shawn | Man | 43 | Professional/scientific/technical services | British Columbia |
| 44 | Theo | Man | 32 | Professional/scientific/technical services | Ontario |
| 45 | Tim | Man | 57 | Transportation and warehousing | British Columbia |
| 46 | Vanessa | Woman | 29 | Educational services | Ontario |
| 47 | Warren | Man | 53 | Construction | Ontario |
